# Intermittent hypoxia changes the interaction of the kinin–VEGF system and impairs myocardial angiogenesis in the hypertrophic heart

**DOI:** 10.14814/phy2.14863

**Published:** 2021-05-15

**Authors:** Bruna Visniauskas, Juliana C. Perry, Guiomar N. Gomes, Amanda Nogueira‐Pedro, Edgar J. Paredes‐Gamero, Sergio Tufik, Jair R. Chagas

**Affiliations:** ^1^ Departamento de Psicobiologia Universidade Federal de São Paulo São Paulo Brazil; ^2^ Departmento de Fisiologia Universidade Federal de São Paulo São Paulo Brazil; ^3^ Departamento de Biofísica Universidade Federal de São Paulo São Paulo Brazil

**Keywords:** bradykinin receptors, carboxypeptidase M, hypertrophy, neovascularization, obstructive sleep apnea, preconditioning, sleep

## Abstract

Intermittent hypoxia (IH) is a feature of obstructive sleep apnea (OSA), a condition highly associated with hypertension‐related cardiovascular diseases. Repeated episodes of IH contribute to imbalance of angiogenic growth factors in the hypertrophic heart, which is key in the progression of cardiovascular complications. In particular, the interaction between vascular endothelial growth factor (VEGF) and the kallikrein‐kinin system (KKS) is essential for promoting angiogenesis. However, researchers have yet to investigate experimental models of IH that reproduce OSA, myocardial angiogenesis, and expression of KKS components. We examined temporal changes in cardiac angiogenesis in a mouse IH model. Adult male C57BI/6 J mice were implanted with Matrigel plugs and subjected to IH for 1–5 weeks with subsequent weekly histological evaluation of vascularization. Expression of VEGF and KKS components was also evaluated. After 3 weeks, in vivo myocardial angiogenesis and capillary density were decreased, accompanied by a late increase of VEGF and its type 2 receptor. Furthermore, IH increased left ventricular myocardium expression of the B2 bradykinin receptor, while reducing mRNA levels of *B1* receptor. These results suggest that in IH, an unexpected response of the VEGF and KKS systems could explain the reduced capillary density and impaired angiogenesis in the hypoxic heart, with potential implications in hypertrophic heart malfunction.

## Graphical abstract

The imbalance between VEGF and B1R‐B2R expressions may contribute to the diminished myocardial angiogenesis in the intermittent hypoxia. Our results indicate a potential role of the reduced capillary density and impaired angiogenesis in the hypertrophic heart with potential sleep apnea implications.

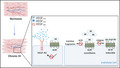

## 1 INTRODUCTION

Obstructive sleep apnea (OSA) is a highly prevalent disorder in the general population and is associated with the development of hypertension‐related cardiovascular diseases (Dewan et al., 2015; Floras, 2018; Nieto et al., 2000). It is characterized by hypoxia episodes due to partial (hypopnea) or total (apnea) obstruction of the upper airways during sleep, followed by reoxygenation, which results in intermittent hypoxia (IH); this consequently leads to hypercapnia, arousals, sympathetic activation, increased blood pressure, and sleep fragmentation (Somers et al., 1995). It has been suggested that repeated episodes of IH, which resemble ischemia/reperfusion events, stimulate adaptive responses, and an imbalance of pro‐ and anti‐angiogenic factors in the hypertrophic heart is key in the progression of cardiovascular complications (Bin‐Jaliah et al., 2010; Dematteis et al., 2009; Park et al., 2007). Imbalanced cardiac angiogenesis and microvascular rarefaction are major contributing factors that aggravate hypertension and its clinical complications such as myocardial ischemia, stroke, and end‐organ damage (Humar et al., 2009). However, while myocardial angiogenesis is an extensively studied subject in the context of ischemia/reperfusion models and sustained hypoxia, there is a lack of information on this process in models of sleep apnea.

Several studies have shown that the interaction between VEGF and the kallikrein‐kinin system (KKS) is essential to the induction of angiogenesis and promotion of cardioprotection under ischemia/reperfusion conditions (Bader, 2009; Emanueli et al., 2001; Sanchez de Miguel et al., 2008). The main active metabolites in the KKS are the kinins bradykinin (BK) and des‐Arg9‐BK; these play crucial roles in vasodilation, the inflammatory process, and vascular remodeling (Regoli et al., 2012). BK and Lys‐BK are, respectively, released from the low and high molecular weight kininogens by plasma and tissue kallikreins. Both are inactivated by angiotensin I‐converting enzyme (ACE), and, respectively, yield des‐Arg9‐BK and des‐Arg10‐Lys‐BK through the action of carboxypeptidase M/N (CPM) (Regoli et al., 2012). The biological effects of des‐Arg9‐BK and BK are, respectively, mediated by type 1 (B1R) and type 2 (B2R) G‐protein coupled receptors. In general, B1R is induced by the action of BK or Lys‐BK on B2R, with B1R activation enhancing the proliferation of endothelial cells from postcapillary venules and promoting angiogenesis (Morbidelli et al., 1998). In primary cultures of cardiac capillary endothelial cells, B2R agonists stimulate endothelial cell growth (mediated by increased VEGF expression) and endothelial NO synthase activation (Harris et al., 2001; Thuringer et al., 2002), indicating that kinins are essential in promoting angiogenesis. Importantly, clinical and animal studies demonstrated that abnormal regulation of B2R‐VEGF, which results in impaired angiogenesis, contributes to increased blood pressure and peripheral resistance, ultimately leading to the development of myocardial hypertrophy and hypertension (Humar et al., 2009).

So far, no investigation has yet been carried out into the correlation of OSA and myocardial angiogenesis with the longitudinal effects of IH. Given this, the importance of the interactions between VEGF and KKS components during ischemic injury led us to inquire as to the role of these same interactions in IH models. Furthermore, a well‐controlled animal model of OSA is essential for demonstrating the short‐ and long‐term impacts of IH on myocardial angiogenesis, the better to clarify the associated mechanisms. Therefore, the goal of the present study was to use an experimental mouse model of apnea to investigate temporal changes in myocardial angiogenesis during IH through histological evaluation of implanted Matrigel plugs and capillary density, along with assessing the left ventricle gene expression of hypoxia inducible factor 1 (HIF‐1), VEGF and its receptor, and KKS components. We hypothesized that in the hypertrophic heart, the myocardial angiogenesis induced in response to IH is reduced through differential expression of VEGF isoforms. Moreover, we hypothesized that imbalance in the expression of VEGF and B1R‐B2R may contribute to diminished angiogenesis and decreased recruitment of endothelial progenitor cells (EPCs). The current findings suggest that impaired VEGF–KKS interaction is responsible for reduction in capillary density and in myocardial angiogenesis. These observations could be essential to understanding the progression of cardiovascular complications in patients with OSA.

## 2 METHODS

### 2.1 Animals

Male C57BL/6 J mice (3–4 months old; weight 20–30 g) were obtained from CEDEME ‐ Universidade Federal de São Paulo (UNIFESP). The animals were housed inside standard polypropylene cages in a colony maintained at 22°C with a 12:12 h light‐dark cycle (lights on at 7:00 AM and off at 7:00 PM). All procedures used in the present study complied with the ARRIVE guidelines and Guide for the Care and Use of Laboratory Animals and were approved by the Ethics Committee of UNIFESP (#1831/11, 2012).

### 2.2 Exposure to intermittent hypoxia (IH)

**FIGURE 1:**
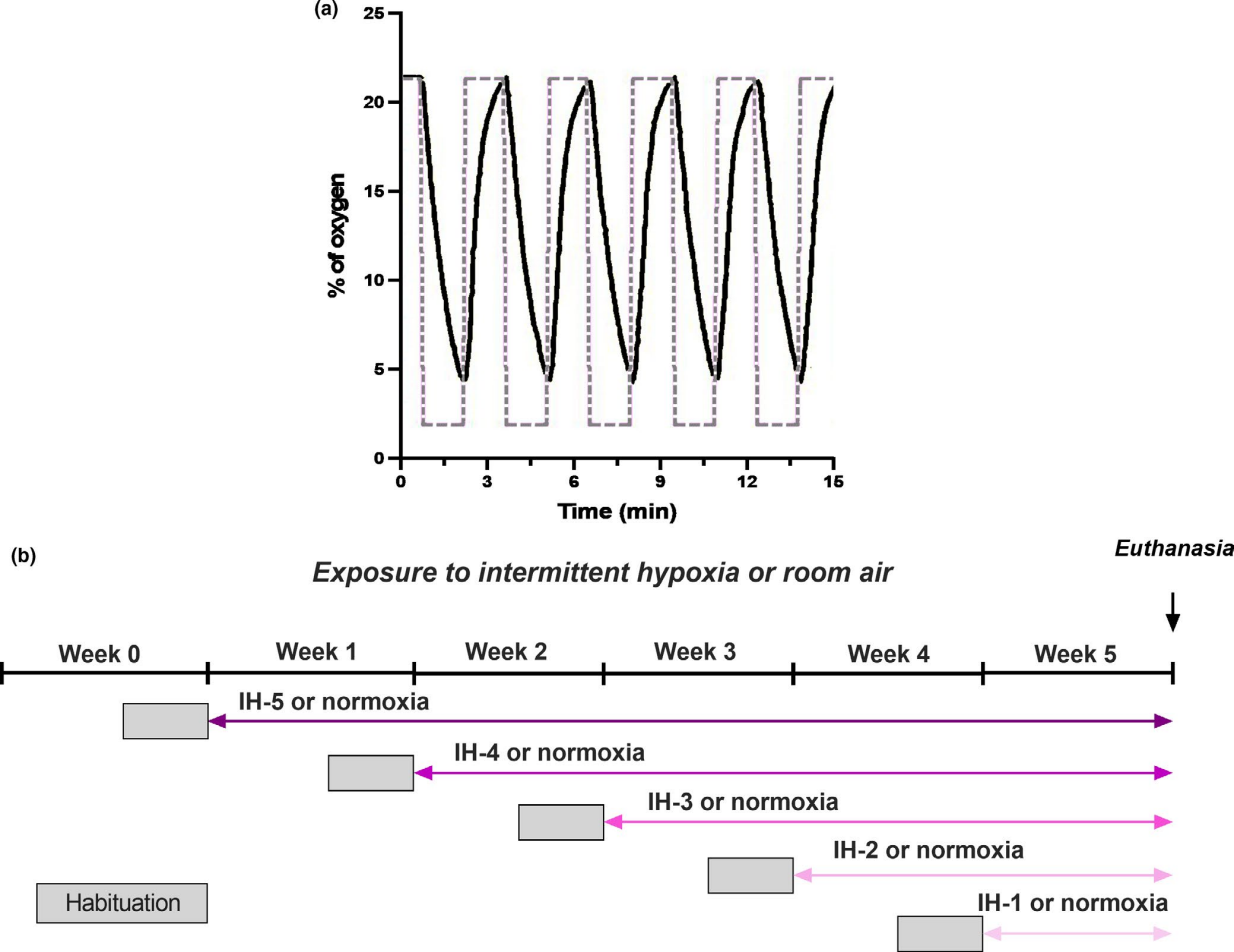
(a) Representative view of the experimental IH protocol. The concentration of oxygen was alternated from 21%–5% in the IH groups. The dashed line represents the setting point used for adjusting O_2_ levels in the chamber. (b) Experimental design of the study design. All animals were habituated three days before to the chamber apparatus and IH protocol (gray square). The animals were subjected to IH or normoxia (control) daily (8 AM–4 PM) for 1, 2, 3, 4, or 5 weeks. Control and IH groups are time‐matched and were euthanized in the same day (B)

The animals were placed in a hypoxia system adapted for mice (Oxycycler model A44XOV, Biospherix, Parish, NY) and exposed to alternating cycles of 21%–5% O_2_. Nitrogen (100%) was distributed into the chamber for approximately 3 minutes to reduce oxygen concentration to 5%. This was followed by air infusion, allowing gradual return (over 15–18 seconds) of ambient air to 21% oxygen. The animals were subjected to 192 episodes of IH per day, 8 h per day (8 AM–4 PM) for 1, 2, 3, 4, or 5 weeks (Figure 1). Carbon dioxide concentration (<0.01%), humidity (40%–50%), and temperature (22–24°C) were monitored during the experiment. Prior to IH treatment, all animals were first habituated three days before to the chamber apparatus and protocol. The respective controls or normoxic animals remained in their home cage under normal oxygen conditions during all weeks of IH treatment (Figure 1B). At the end of each week of treatment, a set of animals were submitted to in vivo myocardial angiogenesis surgery. Another set of animals were euthanized, and the hearts were dissected and weighted. Left ventricle tissues were frozen until the extraction of either RNA or protein. The tibia was removed, and its length recorded.

### 2.3 In vivo myocardial angiogenesis

In order to examine myocardial angiogenesis, we adopted an in vivo protocol previously reported by Zhao and collaborators (Xiang et al., 2009; Zhao et al., 2002). The animals were anesthetized with isoflurane, intubated, and artificially ventilated. After left thoracotomy, the heart was exposed, and 8 µl of Matrigel (Matrigel Matrix high concentration, Becton Dickinson Company, Franklin Lake, NJ) was injected into the wall of the left ventricle near the apex. After three days, the animals were euthanized, and the heart harvested and incubated in fixing solution (10% of formalin) for further histological analysis. Serial sequences (4 μm thick) were cut from the apex to the base of the heart. Hematoxylin and eosin staining were performed for quantification of angiogenesis, which consisted of relating the percentage area of new vessel‐like (or aneurysm‐like) structure penetrating the Matrigel plug to the total Matrigel area (Zhao et al., 2002). Results are presented as the percentage of vessel area. Four random fields of three different sections were visualized by an investigator blinded to the samples using a conventional brightfield microscope (Eclipse 90i, Nikon, Tokyo, Japan), and quantification was performed using ImageJ software (National Institutes of Health, Bethesda, MD).

### 2.4 Immunohistochemistry

Immunohistochemistry was performed to confirm angiogenesis in the Matrigel plug and to quantify capillary density. First, slides were deparaffinized, hydrated, and treated with hydrogen peroxide 0.3% for 15 minutes. The tissues were then blocked (BSA 10%) and incubated at 4°C with anti‐mouse von Willebrand factor (1:200; sc‐365712; Santa Cruz, Dallas, TX) or anti‐mouse CD‐31 for capillary density (1:100; ab9498; Abcam, Cambridge, UK). Immunodetection was performed using the VECTASTAIN Elite ABC Kit (Agilent (Dako), Santa Clara, CA) or MACH 4 (Biocare Medical, Pacheco, CA), and the antigen‐antibody complex was visualized using diaminobenzidine. Slices were examined using a brightfield microscope (Eclipse 50i and 90i, NIS‐Elements software, Nikon, Tokyo, Japan) by an investigator blinded to the samples. Positive microvessel stains were counted in four random fields of three different sections (*n* = 6 hearts per group) using a 40x objective and reported as the number of capillaries per square millimeter.

### 2.5 Quantitative real‐time PCR (RT‐PCR)

Total RNA was isolated from the left ventricular myocardium using the RNeasy Fibrous Tissue kit (Qiagen, Hilden, Germany). Quality and quantity of RNA were confirmed by NanoDrop 8000 spectrophotometer (Thermo Fisher Scientific, Waltham, MA) and ethidium bromide staining in 2% agarose gel. Complementary DNA was synthesized with the High‐Capacity cDNA Reverse Transcription Kit (Thermo Fisher Scientific). Gene expression of *VEGF*, *VEGF*‐*R2*, *HIF*‐*1*, and KKS components in the left ventricle was quantified by TaqMan assay (Thermo Fisher Scientific) on a real‐time PCR system (7500 Thermo Scientific, Life Technologies). The set of customized assays employed to amplify the genes, namely primers/probes and cycle threshold (Ct) values, are described in Table 1. Expression values were normalized using three different endogenous controls (*beta*‐*actin*, *GAPDH*, and *B2 M*), as previously validated by our group for use with the IH protocol (Julian et al., 2016).

**TABLE 1:** Customized TaqMan assays (primers/probes, Thermo Scientific Co, USA), Genbank accession numbers, and mean cycle threshold (Ct) value of the target genes utilized in this study

Genes	Accession number	Customized assay	Mean Ct cDNA 10 μg
*Angiotensin‐converting enzyme 1 (ACE)*	NM_009598.2	Mm00802048_m1	22.9 ± 0.9
*Bradykinin receptor B1 (Bdkrb1)*	NM_007539.2	Mm04207315_s1	31.6 ± 0.4
*Bradykinin receptor B2 (Bdkrb2)*	NM_009747.2	Mm00437788_s1	30.6 ± 0.4
*Carboxypeptidase M (CPM)*	NM_027468.1	Mm01250796_m1	26.1 ± 0.3
*Vascular endothelial growth factor A (VEGF‐A)*	NM_001025250.3	Mm01281449_m1	22.5 ± 0.5
*Vascular endothelial growth factor receptor VEGF‐R2 (kinase insert domain protein receptor – Kdr)*	NM_010612.2	Mm01222421_m1	20.8 ± 0.4
*Hypoxia inducible factor (HIF−1)*	NM_010431.2	Mn00468869_m1	22.4 ± 0.5
*Beta‐actin*	NM_007393.3	Mm02619580_g1	19.4 ± 1.2
*Glyceraldehyde 3‐phosphate dehydrogenase (GAPDH)*	NM_008084.2	Mm99999915_g1	14.6 ± 0.4
*Beta−2 microglobulin (B2 M)*	NM_009735.3	Mm00437762_m1	18.7 ± 0.7

Cycle threshold values are expressed as mean of two technical replicates ± SEM.

### 2.6 ACE and CPM activities in the left ventricle

For protein extraction, left ventricular myocardium tissues were homogenized in Tris‐HCl 50 mM buffer, pH 7.4, containing NaCl 100 mM and Triton X‐100 0.1%. Homogenates were centrifuged at 1,000 *g* for 15 min at 4°C, and the supernatant was frozen at −20°C until assessment of protease activities. Protein content was measured by the Method of Lowry using bovine serum albumin as the standard. ACE activity in left ventricle extracts was determined using the fluorogenic substrate Abz‐FRK(Dnp)‐OH (10 µM) and the specific inhibitor lisinopril at 2 µM final concentration (Alves et al., 2005; Carmona et al., 2006). Assays were performed in a buffer composed of Tris‐HCl 50 mM, pH 7.4, and NaCl 100 mM, with the buffer at 37°C. The reactions were continuously monitored in a fluorometer (Gemini XS, Molecular Devices Company, San Jose, CA), with fluorescence measured at λ_ex_ = 320 nm λ_em_ = 420 nm to determine ACE activity (Carmona et al., 2006). CPM activity was determined using the substrate dansyl‐Ala‐Arg‐OH (100 µM) in Tris‐HCl 50 mM buffer, pH 7.4, with NaCl 50 mM; the specific inhibitor 2‐mercaptomethyl‐3‐guanidinoethylthiopropanoic acid (MGTA, 10 mM) was used during incubations. After 30 minutes of incubation at 37°C, the reaction was stopped using citric acid (1 M), pH 3.1. Then, 500 μl of chloroform was added for extraction of the product dansyl‐Ala‐OH, and an endpoint reading was performed on the fluorometer (λ_ex_ = 340 nm λ_em_ = 495 nm) (Tan et al., 1995). All measurements were performed in triplicate (*N* = 8/group). ACE and CPM activity values are reported as nanomoles of substrate hydrolyzed per minute per milligram of protein (nM.min^–1^.mg^−1^).

### 2.7 Western blotting

The left ventricular myocardium was homogenized in RIPA buffer (Tris‐HCl 100 mM buffer, pH 8.3, containing NaCl 150 mM, sodium deoxycholate 0.5%, EDTA 10 mM, Triton X‐100 1%, SDS 0.1%, glycerol 10% with protease inhibitors (Sigma‐Aldrich, St. Louis, MO). Aliquots of 40 or 60 μg of proteins extracted from the left ventricle were subjected to SDS‐PAGE (10% or 12% gel), transferred to nitrocellulose membrane (Trans‐Blot turbo, Cat. 170–4270, Bio‐Rad, Hercules, CA), and blocked with 3% BSA in phosphate‐buffered saline containing Tween‐20 0.1%. The membrane was incubated with the following primary antibodies: rabbit anti‐VEGF120, 164, and 188 (1:2000; ab51745, Abcam); mouse anti‐VEGF164 (1:2000; ab69479, Abcam); and rabbit anti‐B2R‐Dye680 (1:10,000; Proteimax, Brazil). The anti‐B2R was validated in heart tissue of global knockout mice (B2R‐KO mice shared by Dr. João Bosco of Universidade Federal de São Paulo, Brazil; Supplementary data). Secondary antibody for VEGF was linked with Alexa fluor 680 (1:10,000; Thermo Scientific). Membranes were scanned using the Odyssey Infrared Imaging System (Li‐Cor Biosciences, Lincoln, NE) with appropriate filter sets. The membranes were then stripped and re‐probed (Tris‐HCl 68.5 mM, pH 6.8 with SDS 2%, β‐mercaptoethanol 100 mM, 30 minutes of incubation at 58°C) using glyceraldehyde phosphate dehydrogenase antibody (GAPDH, 1:10,000, G9545, Sigma‐Aldrich) as an internal reference. Secondary antibody for GAPDH was linked with Alexa fluor 680 (1:15,000; Thermo Scientific). Band intensities were quantified using Odyssey 3 software (Li‐Cor Biosciences, Lincoln, NE).

### 2.8 Quantification of endothelial progenitor cells (EPCs)

Peripheral blood was collected in EDTA tubes after decapitation. The blood cells were incubated with lysis buffer (BD Pharm Lyse, Becton Dickinson) according to the manufacturer's protocol. Bone marrow (BM) cells were extracted from the femur cavity using a syringe and needle filled with 3 ml of PBS. The peripheral blood, BM, and heart cells were fixed in paraformaldehyde 2% and immunolabeled with fluorochromes as follows: anti‐CD309/VEGF‐R2‐phycoerythrin (anti‐CD309‐PE; clone QBend‐10; Monosan), anti‐CD45‐allophycocyanin (anti‐CD45‐APC; eBioscience, San Diego, CA), anti‐CD133‐PECy7 (Abcam), and anti‐CD31/PECAM‐BV510 (Becton Dickinson). The phenotypes evaluated were: CD45^−^CD309^+^CD133^−^CD31^+^ (progenitor cells) and CD45^−^CD309^+^CD133^+^CD31^+^ (mature endothelial cells).

A total of 200, 000 progenitor cells were analyzed by flow cytometry on a FACS Aria (Becton Dickinson Bioscience), and data analysis was performed with FlowJo software (Tree Star, Ashland, OR).

### 2.9 Statistical analysis

The Kolmogorov–Smirnov and Bartlett tests were employed to test the normality and homogeneity of the data. All data were analyzed using one‐way ANOVA analysis followed by post hoc Tukey's test (*p* < 0.05 was considered statistically significant). Data are expressed as mean ± standard error of the mean (SEM). Statistical analysis was performed using the software Tibco Statistica v.13.

## 3 RESULTS

No significant differences were observed between the time‐matched normoxic groups for any parameter. Therefore, all normoxic groups were combined in a single cohort for one‐way ANOVA.

### 3.1 Body weight and hypertrophy index

We observed a reduction in mouse body weight during the IH protocol (Table 2; F (4,40) = 7.118; *p* < 0.001). Moreover, left ventricle weights differed significantly between mice receiving 3 and 5 weeks of IH. Normalization of left ventricular weight to tibia length confirmed increase of the left ventricle during intermittent hypoxia (F (5,44) = 5.607; *p* = 0.001).

**TABLE 2:** Body weight and hypertrophy index in mice exposed to IH

	Normoxia	IH−1	IH−2	IH−3	IH−4	IH−5
Body Weight (g)	26.2 ± 0.6	24.7 ± 0.6	22.6 ± 0.7*	22.4 ± 0.5*	23. 0 ± 0.3*	23.9 ± 0.4*
Left ventricle (mg)	75 ± 4.0	85 ± 6.8	74.3 ± 3.1	100.1 ± 3.1*	86 ± 5.3	97.7 ± 7.1*
LV/Tibia length (mg/mm)	4.16 ± 0.2	4.37 ± 0.2^#^	4.16 ± 0.16#	5.8 ± 0.28*	5.15 ± 0.25*	5.42 ± 0.42*
Tibia length (mm)	17.8 ± 0.1	18.2 ± 0.1	17.8 ± 0.2	17.8 ± 0.2	17.5 ± 0.1	18.0 ± 0.1

Note

Body weight: *different from normoxia (*p* < 0.05). Left ventricle: *different from normoxia (IH‐3: *p* = 0.018; IH‐5: *p* = 0.04). Left ventricle/tibia length ratio *different from normoxia (IH‐3: *p* = 0.003; IH‐5: *p* = 0.04) #different from IH‐3 (IH‐1: *p* = 0.02; IH‐2: *p* = 0.005). N = 10‐15/group. Results are expressed as mean ± SEM.

### 3.2 Myocardial angiogenesis is decreased in the left ventricle of mice exposed to intermittent hypoxia

**FIGURE 2:**
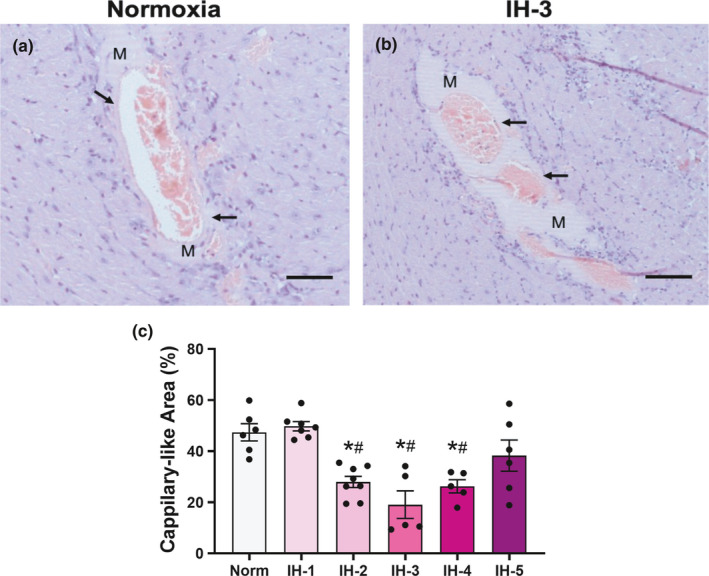
*In vivo* myocardial angiogenesis. Representative hematoxylin/eosin staining sections of the left ventricle from a normoxia and IH‐3 (a and b). Representative images were obtained using a 20x objective. Arrows indicate the occurrence of cardiac angiogenesis around the Matrigel plug. M indicates the Matrigel plug in the left ventricular myocardium. (c) Quantitative analysis of angiogenesis, *Different from normoxia (*p* < 0.001), #different from IH‐1 (*p* < 0.001), *N* = 6–8/group. Results are expressed as the percentage of vessel area in the total Matrigel area, mean ± SEM. Scale bar: 100 μM

**FIGURE 3:**
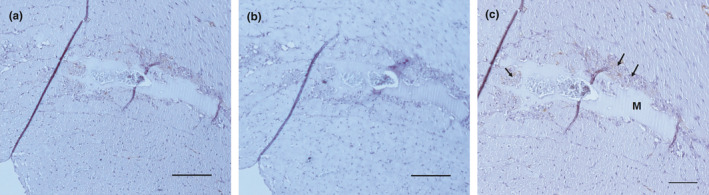
Immunohistochemistry for von Willebrand factor in the left ventricle of mice exposed to 2 weeks of IH. Representative microphotographs were obtained using 10x objective (*n* = 2–3/per group) a and b. Matrigel (M) was implanted in the left ventricle myocardium. Scale bar: 200 μM. (b) Negative control. (c) Representative immunohistochemistry images of Von Willebrand in left ventricle (20 x objective). Arrows indicate the positive Von Willebrand stained around the Matrigel plug. Scale bar: 50 μM

**FIGURE 4:**
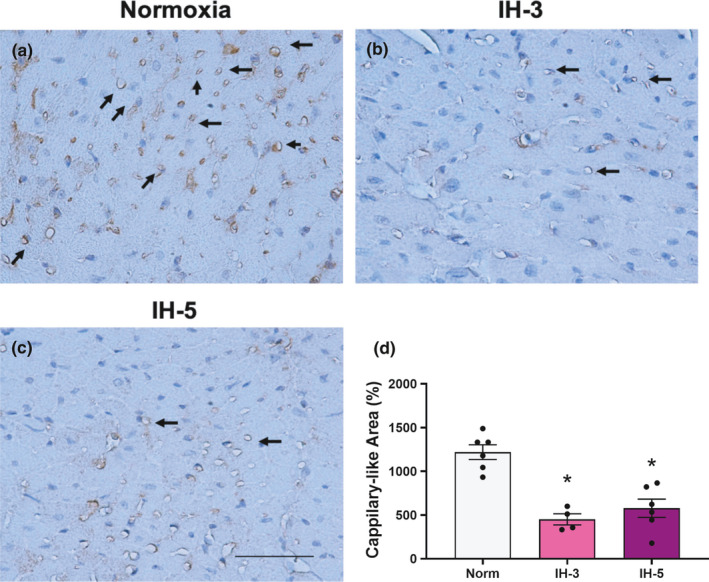
Capillary density. CD31/PECAM staining in heart sections of mice under hypoxia intermittent. Representative microphotographs were obtained using a 40x objective (a, b, c). Arrows indicate the CD‐31/PECAM stained in the left ventricle. (d) *different from normoxia (*p* < 0.001). *N* = 4–6/group. Data mean ±SEM. Scale bar: 50 μm

We first examined in vivo myocardial angiogenesis in animals exposed to IH, obtaining representative microphotographs using a 20x objective (Figure 2A and 2B). The newly formed vessels that penetrated the Matrigel plug were surrounded by inflammatory cells and aneurysm structures. In mice receiving 2, 3, and 4 weeks of IH, myocardial angiogenesis was diminished relative to the normoxia group and IH‐1 (F (5,31) = 10.94, *p* < 0.001, Figure 2C). Occurrence of cardiac angiogenesis around the Matrigel is indicated by cells positive for the von Willebrand factor being present around the new (capillary‐like) structure (Figure 3). In addition, capillary densities in the heart were decreased in mice receiving 3 and 5 weeks of intermittent hypoxia when compared with normoxic mice (F (2,13) = 20.33; *p* < 0.001, Figure 4).

### 3.3 Expression of angiogenic factors and relative quantification of endothelial progenitor cells (EPCs) under intermittent hypoxia

**FIGURE 5:**
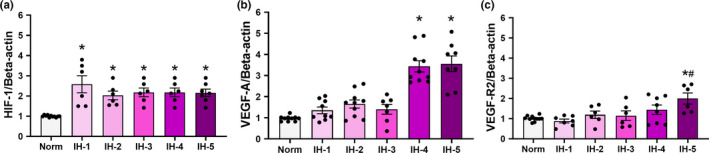
Angiogenic factors in left ventricular myocardium by duration of intermittent hypoxia. Plot of relative amounts of *HIF*‐*1* (a) *VEGF*‐*A* (b) and *VEGF*‐*R2* (c) mRNA determined by RT‐PCR (*N* = 8–12/group). (a) *Different from normoxia (*p* = 0.001). (b) *Different from normoxia (*p* < 0.001). (c) *Different from normoxia (*p* = 0.001); # different from 1 week of IH (*p* = 0.02). Data are mean ± SEM

We evaluated temporal changes in the expression of angiogenic factors in the left ventricular myocardium by RT‐PCR and Western blotting, specifically for HIF‐1, VEGF, and VEGF‐R2. Figure 5 shows *HIF*‐*1*, *VEGF*‐*A*, and *VEGF*‐*R2* mRNA expression normalized to that of endogenous *beta*‐*actin*. Normalization to endogenous expression of the genes *GAPDH* and beta‐2‐microglobulin (*B2 M*) yielded similar results (Table 3). As expected, *HIF*‐*1* gene expression increased at the first week of IH and remained elevated through all five weeks of IH (F (5,36) = 8.611, *p* < 0.0001, Figure 5A). However, *VEGF*‐*A* expression was increased only in the fourth week of IH (F (5,48) = 26.06, *p* < 0.001; Figure 5B), and *VEGF*‐*R2* expression increased only in the fifth week of IH (F (5,38) = 4.754, *p* = 0.001, Figure 5C).

**TABLE 3:** Expression values using *GAPDH* and *B2 M* endogenous genes

	Normoxia	IH−1	IH−2	IH−3	IH−4	IH−5
*HIF−1*						
*GAPDH*	1.0 ± 0.05	2.4 ± 0.3	3.0 ± 0.4*	2.6 ± 0.5*	3.0 ± 0.6*	2.3 ± 0.2*
*B2 M*	0.95 ± 0.1	1.6 ± 0.2	2.5 ± 0.5*	2.0 ± 0.6	2.6 ± 0.4*	1.7 ± 0.2
*VEGF‐A*						
*GAPDH*	1.0 ± 0.1	2.1 ± 0.4	2.0 ± 0.3	1.6 ± 0.2	2.6 ± 0.3*	2.6 ± 0.1*
*B2 M*	1.0 ± 0.1	1.7 ± 0.2	2.6 ± 0.3	2.5 ± 0.8	3.2 ± 0.5*	2.1 ± 0.6
*VEGF‐R2*						
*GAPDH*	1.0 ± 0.2	0.9 ± 0.1	2.1 ± 0.3	3.2 ± 0.6	3.2 ± 0.7	2.9 ± 0.6*
*B2 M*	1.0 ± 0.1	0.78 ± 0.1	1.3 ± 0.2	1.7 ± 0.4	1.9 ± 0.1	2.4 ± 0.7*
*B2R*						
*GAPDH*	1.0 ± 0.2	1.6 ± 0.2	1.3 ± 0.1	2.1 ± 0.4*	1.2 ± 0.08	1.1 ± 0.1
*B2 M*	0.97 ± 0.1	1.7 ± 0.1	2.9 ± 0.3*	2.5 ± 0.3*	2.3 ± 0.3*	1.4 ± 0.2
*ACE*						
*GAPDH*	1.0 ± 0.1	1.3 ± 0.1	1.5 ± 0.1	2.2 ± 0.4*	1.2 ± 0.1	0.75 ± 0.1^§^
*B2 M*	0.9 ± 0.1	1.0 ± 0.1	1.4 ± 0.1*#	1.6 ± 0.1*	1.2 ± 0.05	0.63 ± 0.1^§^
*CPM*						
*GAPDH*	1.0 ± 0.1	0.86 ± 0.1	1.2 ± 0.1	1.1 ± 0.1	0.95 ± 0.04	0.83 ± 0.07
*B2 M*	1.1 ± 0.1	0.94 ± 0.2	1.7 ± 0.2*#	1.4 ± 0.2	1.6 ± 0.2	1.3 ± 0.2

Note

*HIF*‐*1*, *GAPDH*: F (5,35) = 2.5782, *p* = 0.04, *different from normoxia (*p* = 0.04). *B2 M*: F (5,30) = 36,215, *p* = 0.011, *different from normoxia (*p* = 0.014). *VEGF*, *GAPDH*: F (5,32) = 26,461, *p* = 0.04, *different from normoxia (*p* = 0.04). *B2 M*: F (5,33) = 26,461, *p* = 0.04, *different from normoxia (*p* = 0.04). *VEGF*‐*R2*, *GAPDH*: F (5, 29) = 40,055, *p* = 0.001, *different from normoxia (*p* = 0.03). *B2 M*: F (5, 33) =26,461, *p* = 0.05. *different from normoxia (*p* = 0.04) and IH‐1 (*p*‐0.04). *B2R*, *GAPDH*: F (5, 37) = 30,874, *p* = 0.02, *different from normoxia and IH‐5 (*p* = 0.035). *B2 M*: F (5, 32) = 39,749, *p* = 0.006 *different from normoxia (*p* = 0.013); *ACE*, *GAPDH*: F (5, 37) =59,314, *p* < 0.001. *different from normoxia (*p* = 0.011), IH‐1 (*p* = 0.03), IH‐4 (*p* = 0.015), and IH‐5 (*p* = 0.0001). *B2 M*: F (5, 28) = 12,436, *p* = 0.0001. *different from normoxia (*p* = 0.01) and § different from IH‐1 (*p* = 0.02); # different from IH‐2 (*p* = 0.001) and IH‐3 (*p* = 0.001). *CPM*, *GAPDH*: F (5, 37) = 22,289, *p* = 0.07. *B2 M*: F (5, 33) = 24,485, *p* = 0.05. *different from normoxia (*p* = 0.04); §different from IH‐1 (*p* = 0.03).

**FIGURE 6:**
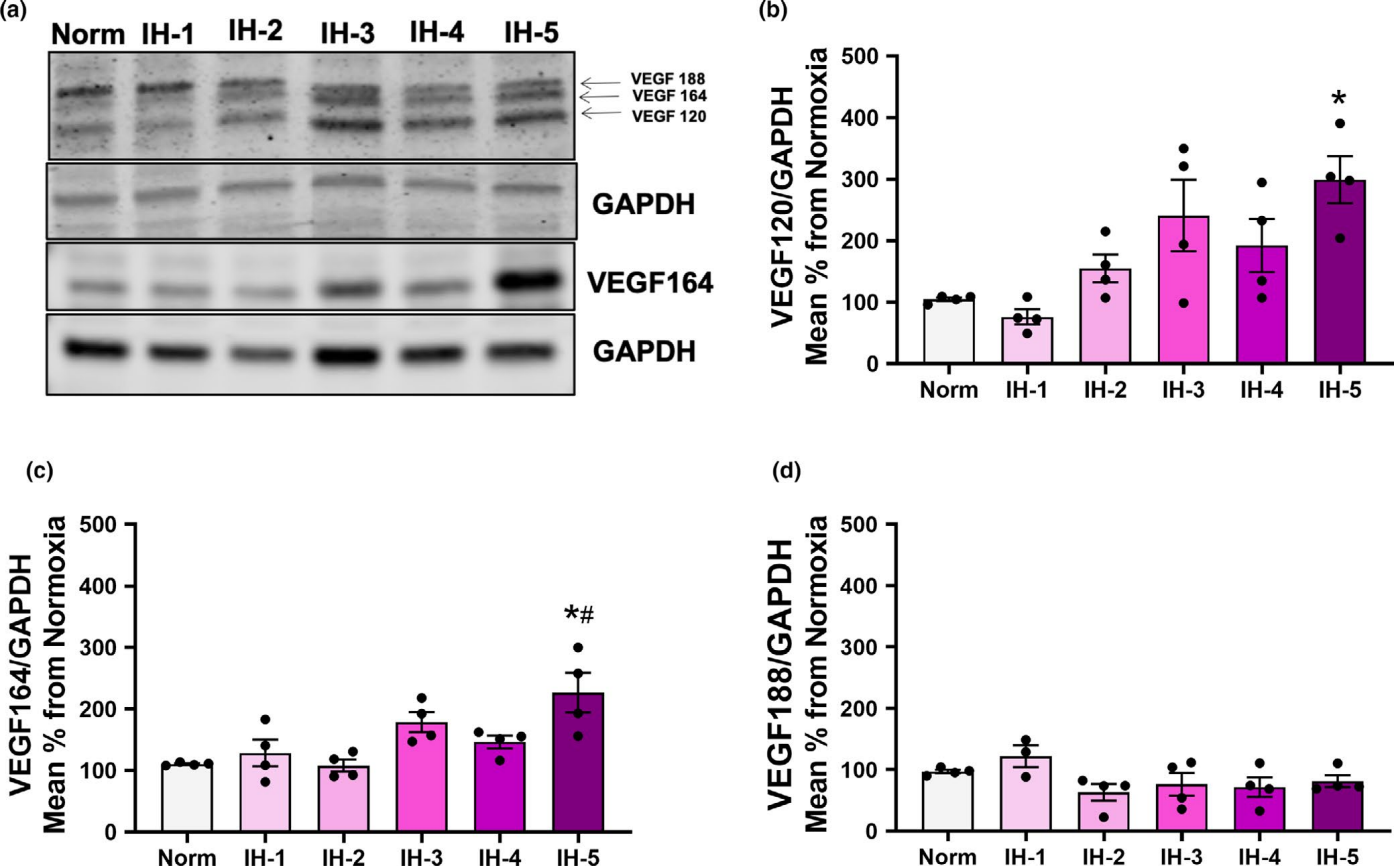
Protein expression of VEGF isoforms in left ventricular myocardium by duration of intermittent hypoxia. (a) Representative images of cardiac VEGF_188_, VEGF_164_, and VEGF_120_ in the left ventricle quantified by Western blot with normalization against GAPDH (*N* = 4/group). (b) Densitometry analysis of VEGF_120_. *different from normoxia (*p* = 0.01) and IH‐1 (*p* = 0.03). (c) Densitometry analysis of VEGF_164_. *different from normoxia (*p* < 0.001) and IH‐1 (*p* = 0.01). (d) Densitometry analysis of VEGF_188,_ no significant changes found. Results are expressed as mean ± SEM in arbitrary units

In protein expression analysis, the anti‐VEGF antibody from Abcam detects VEGF_188_, VEGF_164_, and VEGF_120_ isoforms in the mouse heart (Figure 6A). However, we chose to only quantify VEGF_188_ and VEGF_120_ with this antibody on account of the spacing between the bands and greater reliability in the results; we used a different antibody for the quantification of VEGF_164_. Under hypoxia, VEGF_120_ expression increased in the left ventricle (F: (5,47) = 2.631, *p* = 0.03, Figure 6B), while VEGF_188_ remained unchanged (F (5,35) = 7.488, *p* < 0.001, Figure 6D). Figure 6C shows an increase in VEGF_164_ expression in the fifth week of IH (F (5, 40) = 17.91, *p* < 0.001, Figure 6C).

To demonstrate an additional mechanism that could explain the impaired angiogenesis in IH, we used flow cytometry to quantify EPCs and mature endothelial cells in the blood, bone marrow, and heart. Progenitor cells were defined by the phenotype CD45^−^CD309^+^CD133^−^CD31^+^, and mature endothelial cells by the phenotype CD45^−^CD309^+^CD133^+^CD31^+^. As depicted in Table 4, the percentages of EPCs and mature endothelial cells in the bone marrow increased in the third week of IH (EPCs: F (5,23) = 3.354; *p* = 0.02; mature endothelial cells: F (5,24) = 5.384, *p* = 0.001). Similarly, an increase in EPCs was observed in the heart at the third week of IH (F (5,15) = 4.570, *p* = 0.001). No changes were observed for the percentages of EPCs and mature endothelial cells in the blood at any time point.

**TABLE 4:** Quantification of endothelial progenitor cells and mature endothelial cells in intermittent hypoxia

	Normoxia	IH−1	IH−2	IH−3	IH−4	IH−5
*Bone Marrow*						
%EPC	0.017 ± 0.001	0.005 ± 0.001	0.0140 ± 0.003	0.0410 ± 0.007*#	0.0156 ± 0.002	0.0216 ± 0.008
% of Mature endothelial cells	1.25 ± 0.19	0.73 ± 0.12	1.55 ± 0.12	2.74 ± 0.54*#	1.21 ± 0.29	1.57 ± 0.58
*Peripheral Blood*						
%EPC	0.36 ± 0.10	0.91 ± 0.32	0.97 ± 0.60	0.70 ± 0.18	0.24 ± 0.08	0.77 ± 0.46
% of Mature endothelial cells	0.88 ± 0.30	1.97 ± 0.68	1.78 ± 0.86	0.98 ± 0.29	0.86 ± 0.28	1.11 ± 0.43
*Heart*						
%EPC	0.004 ± 0.002	0.007 ± 0.002	0.007 ± 0.003	0.020 ± 0.004*#	0.015 ± 0.002	0.003 ± 0.001
% of Mature endothelial cells	0.030 ± 0.010	0.008 ± 0.007	0.008 ± 0.002	0.022 ± 0.007	0.017 ± 0.007	0.016 ± 0.005

Note

In the bone marrow, % EPCs and % mature endothelial cells were *different from normoxia (*p* = 0.01), and #from IH‐1 (*p* = 0.001). In the heart, % EPCs were *different from normoxia (*p* = 0.01), and #from IH‐1 (*p* = 0.001). N = 5–6/group. Results are expressed as mean ± SEM.

### 3.4 Time course of changes in ventricular expression of KKS components under intermittent hypoxia

**FIGURE 7:**
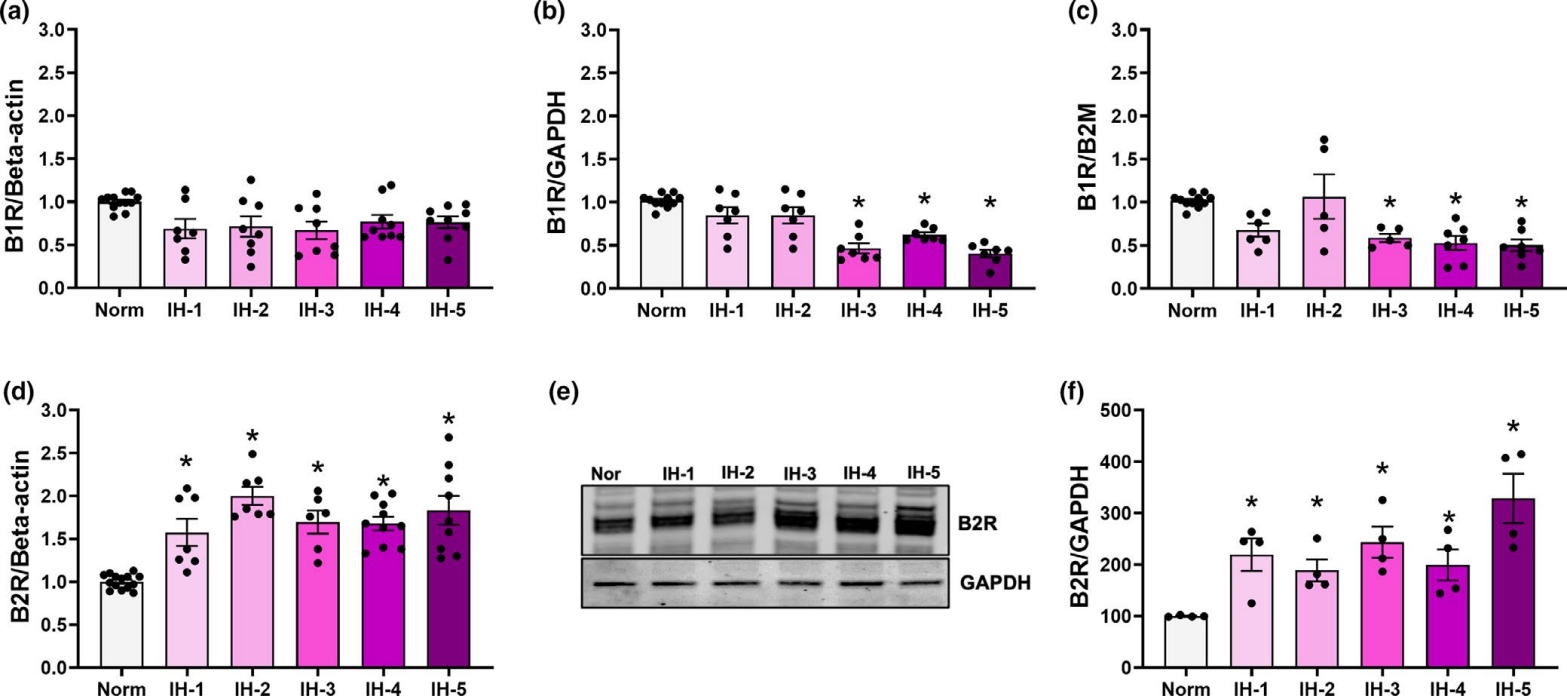
*B1R* mRNA levels and B2R expression in the left ventricular myocardium by duration of intermittent hypoxia. Plot of *B1R* mRNA levels relative to β‐actin (a) *GAPDH* (b) and *B2 M* (c) as endogenous controls (*N*=8‐12/group). (b) *Different from normoxia (*p* < 0.001). (c) *Different from normoxia (*p* < 0.02). (d) Plot of *B2R* mRNA expression as determined by RT‐PCR (N = 8–12/group). *different from normoxia (*p* < 0.001). (e) Representative western blot of B2R protein expression in the left ventricle (*N* = 4/group). (f) Densitometry analysis of B2R, normalized against GAPDH. *different from normoxia (*p* = 0.03). Results are expressed as mean ± SEM

To determine temporal changes in KKS components during IH, we investigated the expression of *B1R*, *B2R*, *ACE*, and *CPM*, along with the activities of the latter two proteins. Relative to controls, *B1R* mRNA expression in the left ventricle was decreased in the third week of IH and remained reduced at the end of five weeks of IH (*B1*/*GAPDH*: F (5,40) = 17.91; *p* < 0.001; *B1*/*B2 M*: F (5, 35) = 7.488, *p* < 0.001, Figure 7). A similar tendency was observed using beta‐actin as an endogenous control for gene expression analysis (*B1R*/*beta*‐*actin*: F (5,47) = 2.631, *p* = 0.14, Figure 7A).

**FIGURE 8:**
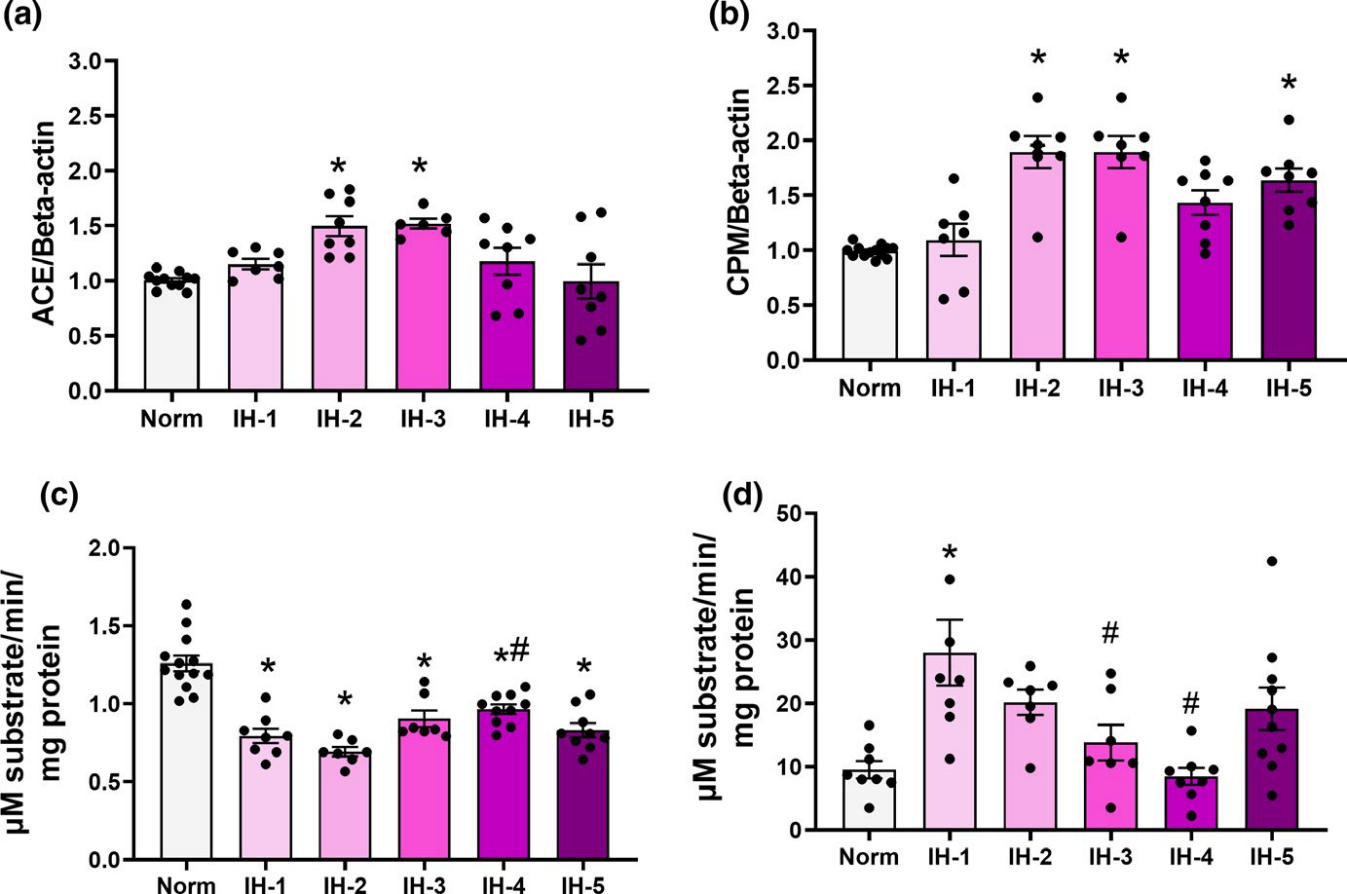
*ACE* and *CPM* gene expression and protein activity in left ventricular myocardium by duration intermittent hypoxia. Plot of *ACE* (a) and *CPM* (b) mRNA expression as determined by real‐time PCR (*N* = 8–12/group). ACE (c) and CPM (d) activities (*N* = 8–14/group). (a) *Different from normoxia (*p* = 0.04). (b) *different from normoxia (*p* = 0.012). (c) *Different from normoxia (*p* < 0.001), # different from 2 weeks of IH (*p* = 0.002). (d) *Different from normoxia (*p* = 0.001), # different from 1 week of intermittent hypoxia: IH‐1 vs. IH‐3, *p* = 0.03; IH‐1 vs. IH‐4, *p* < 0.001. Data are mean ± SEM

In contrast, *B2R* mRNA and protein expression in the left ventricle were significantly higher from the first week of IH (Figure 7D, 7E, and 7F). At the end of five weeks of IH, *B2R* gene and protein expression both remained elevated (gene expression: F (5,47) = 13.83, *p* < 0.001, Figure 7D; protein expression: F (5,18) = 6.018, *p* = 0.001, Figure 7F). Meanwhile, *ACE* and *CPM* genes showed an increase in gene expression after two weeks of IH (ACE: F (5,42) = 7.331, *p* < 0.001, Figure 8A, CPM: F (5,45) = 13.95; *p* < 0.001, Figure 8B). Using specific substrates and inhibitors, we then evaluated ACE and CPM activities in the total left ventricle extracts of mice exposed to IH for one or more weeks. ACE activity in the left ventricular myocardium was decreased in the first week of IH, and remained reduced at the end of five weeks’ treatment (F (5,48) = 21.98; *p* < 0.001, Figure 8C). In contrast, CPM activity in the left ventricle increased during the first week of hypoxia (F = (5,42) = 5.672; *p* < 0.001, Figure 8D), but returned to values consistent with the control group afterward.

## 4 DISCUSSION

The present study provides evidence on the time course evolution of myocardial angiogenesis, capillary density, angiogenic factors, and KKS components during five successive weeks of IH exposure. Myocardial angiogenesis and capillary density are both decreased from the third to the fifth weeks, accompanied by delayed increase in both gene and protein expression of VEGF. Furthermore, 3 weeks of IH induced cardiac hypertrophy and increased abundance of bone marrow EPCs and mature endothelial cells, along with an opposing change in *B1R* mRNA levels. *B2R* mRNA and protein expression remained elevated through the entire exposure course. Interestingly, ACE activity was reduced, despite an increase in gene expression. However, CPM expression and activity were increased by the first and second weeks of IH, returning to control levels in subsequent weeks. These results suggest that in IH, unlike in other models of hypoxia, the classic interaction between VEGF and KKS components and their temporal expression are not sufficient to stimulate the formation of new vessels. Our following discussion will cover the role of the impaired angiogenesis resulting from the delayed increase of VEGF isoforms, along with the importance of the VEGF–KKS interaction during IH.

### 4.1 Left ventricular hypertrophy, impaired myocardial angiogenesis, and capillary density during IH

Intermittent hypoxia is highly associated with left ventricular hypertrophy in patients with sleep apnea and rodent models (Campen et al., 1985; Noda et al., 1995; Yamaguchi et al., 2016). A previous report demonstrated that the significant increases in left ventricle weight that occurred in response to acute and chronic IH were only evident after adjustment for body weight (Campen, Shimoda, et al., 1985). Weight loss during severe IH (5% oxygen) is known to be related to increased circulating leptin and insulin resistance (Breslow et al., 1999; Drager et al., 2011; Li et al., 1985). We observed significantly decreased body weight and increased left ventricular mass, verified with reference to tibia length. The fact that the presence of left ventricular hypertrophy in response to IH has remained controversial in some studies (Campen, Shimoda, et al., 1985), more studies including a detailed analysis of cardiac architecture are needed to better define the heart hypertrophy impact in IH complications, particularly with hypoxic C57Bl/6 J mice.

Reduced cardiac angiogenesis is a common feature of adaptive cardiac hypertrophy and contributes to its progression to heart failure (Gogiraju et al., 2019). In addition to impaired angiogenesis, hypertrophy of the heart is frequently associated with decreased myocardial capillary density (Choi et al., 2008), and elevation of blood pressure (Humar et al., 2009). According to Dematteis et al., mice exposed to two and five weeks of IH demonstrate decreased endothelial CD‐31/PECAM‐1 expression in the descending aorta and left heart (Dematteis et al., 2008). This is consistent with our observations, where in the third week of IH HIF‐1 activity was increased, no change in VEGF protein was evident, and capillary density was reduced in the hypertrophied myocardium (Choi et al., 2008). Therefore, a possible partial explanation for the impaired left ventricular angiogenesis in IH could be an inability to increase the number of capillaries in the myocardium, as demonstrated in the present study. Consistent with this hypothesis, reductions in overall capillary density and VEGF expression were identified in patients with cardiomyopathy and heart hypertrophy (Gogiraju et al., 2019). Collectively, as suggested by clinical and experimental models, impaired angiogenesis and cardiac capillary density contribute to the transition from hypertrophy to heart failure.

### 4.2 Delayed increases in VEGF isoform expression and decreased recruitment of EPCs contribute to impaired angiogenesis

It is well established that HIF‐1, VEGF, and VEGF‐R2 are critical components in the induction and maintenance of angiogenesis (Olsson et al., 2006). The observed increase of *HIF*‐*1* in the myocardium of rodents under IH was expected (Wang & Si, 2013). We also observed delayed increases of *VEGF*‐*A* transcript levels, VEGF_164_ and VEGF_120_ isoforms with long‐term IH, whereas VEGF_188_ expression was unchanged. A few studies have shown that in the setting of post‐myocardial infarction and diabetic cardiomyopathy, VEGF isoforms differ in their tissue‐specific expression, expression patterns, and tissue kinetics (Han et al., 2009; Zhao et al., 2010). A mouse model in which VEGF_164_ and VGF_188_ isoforms were deleted and solely VEGF_120_ was expressed demonstrated that impaired myocardial angiogenesis leads to ischemic cardiomyopathy. Therefore, VEGF angiogenic activity is dependent on the respective levels of its isoforms (Carmeliet et al., 1999). Our data indicate that during five weeks of IH, the observed late increase of VEGF_121_ and VEGF_165_ is insufficient to stimulate angiogenesis.

VEGF also plays a critical role in the mobilization of EPCs to promote angiogenesis, endothelium maintenance, and vascular repair in the ischemic heart (Szmitko et al., 2003). In patients with myocardial infarction, those also having sleep‐disordered breathing present higher levels of circulating VEGF and increased proliferative and angiogenic capacity of EPCs (Berger et al., 2013; Berger & Lavie, 2011). In the present study, EPCs and mature endothelial cells were significantly more abundant in both heart and bone marrow by the third week of IH, which changes parallel the augmentation of VEGF in that same period. Thus, increased VEGF appears to favor a late increase in the recruitment of EPCs from bone marrow to restore angiogenesis and blood flow in the hypoxic heart.

### 4.3 Interaction between VEGF and KKS in IH

The interaction between VEGF and bradykinin receptors is essential in promoting angiogenesis (Humar et al., 2009). Our results suggest that in IH, an unexpected response of the VEGF and KKS systems could explain the reduced capillary density and impaired angiogenesis in the hypoxic heart; this has potential implications in hypertrophic heart malfunction. In a mouse heart organ culture model under sustained hypoxia (1% O_2_), BK receptors positively regulated the increase of VEGF and promoted angiogenesis (Sanchez de Miguel et al., 2008). In addition, VEGF‐R2 is a tyrosine kinase receptor and B2R a G‐coupled protein receptor, they have been described as having common signaling cascades and crosstalk. (Miura et al., 2003; Thuringer et al., 2002). This raises the prospect that B2R and VEGF‐R2 may functionally interact to modulate downstream effects and drive angiogenesis. However, in our model, B2R increased from the first week of IH and remained elevated, whereas *VEGF*‐*A and VEGF*‐*R2* transcript levels increased only in the fourth week. Nonetheless, the elevation in B2R levels and late increase of VEGF‐A and VEGFR‐2 in the present IH exposure course were not sufficient to stimulate angiogenesis.

Under myocardial ischemic injury, B2R activation is essential for the upregulation of *B1R* expression (Spillmann et al., 2002; Tschope et al., 2000). While elevated levels of HIF‐1 and B2R were observed throughout the present IH exposure course, angiogenesis was decreased, which could be linked to the reduction of or absence of an increase in *B1R* levels. B1R activation is essential in reversing the consequences of ischemia in the heart (Alhenc‐Gelas et al., 2011; Chahine et al., 1993; Su, 2006; Tschöpe et al., 2004). Similarly, interaction between B1R and B2R in the plasma membrane seems to favor this effect (Kang et al., 2004). CPM is also a positive allosteric regulator of B1R (Zhang et al., 2013). In our study, CPM increase was observed only in the first week of IH, with reversion to control levels in subsequent weeks, while *B1R* gene expression underwent an evident decrease. Thus, lower *B1R* expression could explain the reduced angiogenesis. In support of this hypothesis, absence of *B1R* in microvascular endothelial cells was reported to downregulate CPM expression and activity (Guimaraes et al., 2019) and to impair angiogenesis in animals submitted to hind limb ischemia (Emanueli et al., 2001). In the same sense, B1R activation is responsible for increased *CPM* transcript expression, protein expression, and activity (Guimaraes et al., 2019), and for inducing proliferation and favoring angiogenesis in endothelial cells (Regoli et al., 2012). Finally, B1 bradykinin receptor is required for the FGF‐2 upregulation that induces VEGF expression in the endothelial cells of forming capillaries (Parenti et al., 2001; Seghezzi et al., 1998). Taken together, these data indicate a meaningful role of B1R in cardiac angiogenesis and highlight the absence of change in *B1R* as a potential explanation for our results.

**FIGURE 9:**
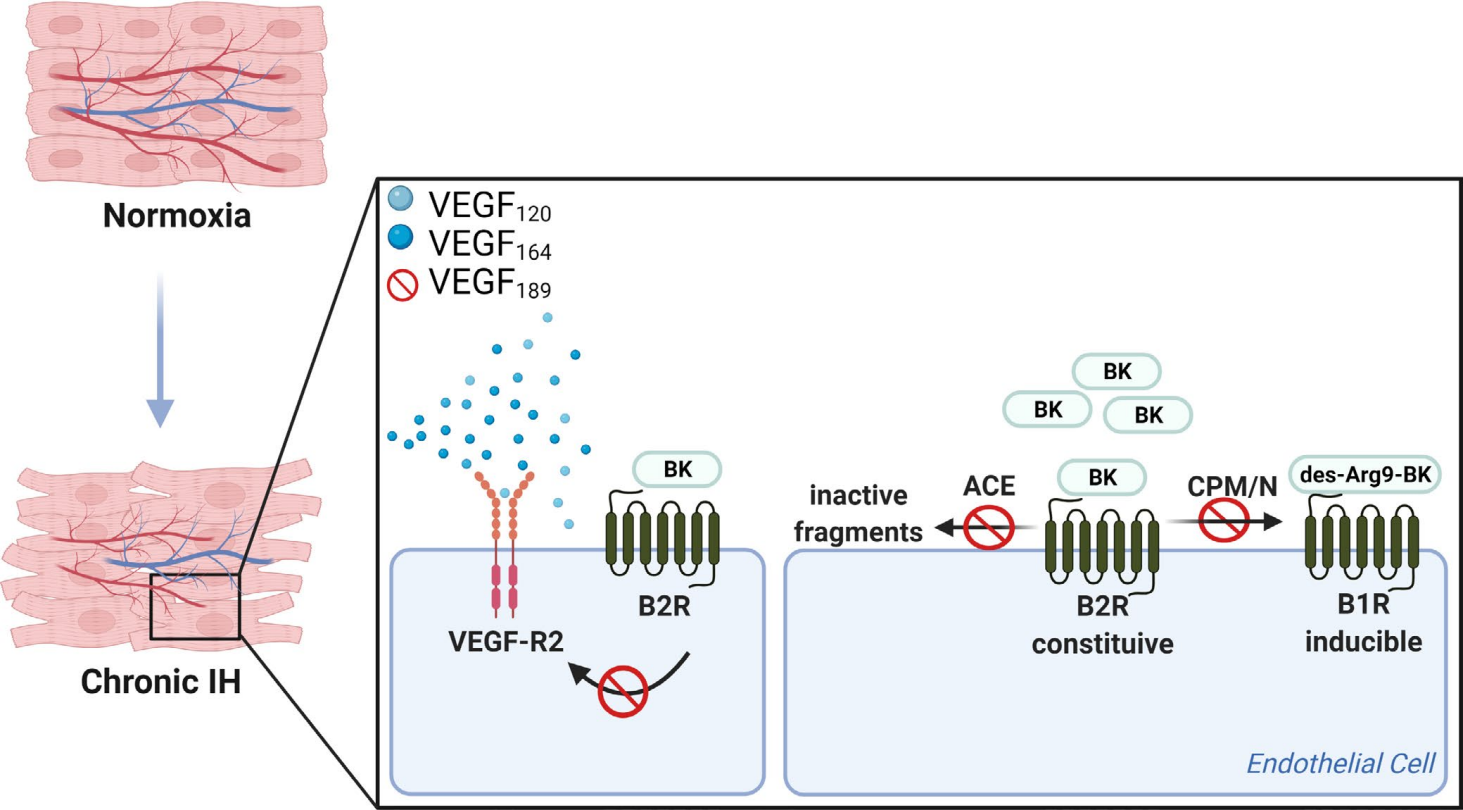
Hypothetical scheme demonstrating possible consequences in the hypoxic heart. Possible effects of delayed increase of VEGF isoforms and abnormal interaction of the VEGF–KKS in 5 weeks of IH. Decreased ACE activity would increase the local concentration of BK, which in turn stimulates VEGF and interactions with B2R and VEGFR‐2. Furthermore, reduction of B1R downregulates CPM expression and activity in endothelial cells. Therefore, as demonstrated in the present study, the abnormal regulation of B2R/B1R‐VEGF‐R2 may contribute to impaired angiogenesis. Illustration created with BioRender.com

ACE is also relevant in the control of angiogenesis, as ACE inhibitors have been shown to induce angiogenesis (Sanchez de Miguel et al., 2008). In the present study, we document the same effect previously described for a rat infarction model, where ACE mRNA expression was increased in heart and lung tissues while its protein activity was decreased (Atochina et al., 1997; Gaertner et al., 2002). Ischemia and reperfusion are known to accelerate the shedding of ACE from the pulmonary endothelium (Atochina et al., 1997). As a membrane‐anchored protein, the apparent lack of correlation between ACE mRNA level and protein activity could reflect a different turnover rate for membrane‐bound ACE in the heart endothelium. It is reasonable to expect that a decrease of ACE activity over time would increase the local concentration of BK, which in turn stimulates VEGF and interactions with B2R and VEGFR‐2. However, as demonstrated in the present study, the abnormal regulation of B2R‐VEGF‐R2 may contribute to impaired angiogenesis (Figure 9). Significantly, abnormal regulation of B2R‐VEGF/VEGF‐R2 and impaired angiogenesis contributes to increased peripheral resistance and blood pressure, leading to hypertension and end‐organ damage (Humar et al., 2009), as observed in OSA.

### 4.4 IH protocol considerations and limitations of the study

It is well established that the transcription factor HIF‐1 is regulated by hypoxia and plays an essential role in regulation of VEGF expression. Unexpected results of the current study included a sustained increase of *HIF*‐*1* mRNA after the first week, an increase in *VEGF*‐*A* at the fourth week, and no evident trend change in myocardium angiogenesis. Notably, HIF‐1 activity is not only regulated by hypoxia, and once activated, it may amplify oxidative stress via mitochondrial ROS generation (Semenza, 2009). The sustained activation of HIF during IH is due to oxidative stress produced by the cycles of oxygenation and desaturation (Verges et al., 2015). Whether *HIF*‐*1* expression increases immediately in the first week of IH and why it appears to not regulate *VEGF* transcript levels in this context is unclear and requires further investigation. Despite an increase in *VEGF*‐*A mRNA*, we could only detect an increase in *VEGF*‐*A* protein expression at the fifth week and *VEGF*‐*R2* transcript levels in the fourth week of IH. Regulatory elements may interact in the myocardium during IH to promote post‐translational modifications; it is reasonable that the protein expression of VEGF and its receptor protein differ from RNA transcript levels and contribute to the reduced angiogenesis (Carmeliet et al., 1999).

Several studies have proposed that IH activates adaptive cardioprotective responses elicited by preconditioning, and furthermore that HIF‐1 and its target genes contribute to these responses (Beguin et al., 1985; Verges et al., 2015; Wang & Si, 2013). In particular, increase of VEGF in the heart during sustained hypoxia or ischemia/reperfusion may protect from damage through promoting angiogenesis (Bin‐Jaliah et al., 2010; Dematteis et al., 2009). The apparent delay of angiogenesis to the fifth week of IH may indicate late adaptive responses. Although our protocol elicits detrimental effects in terms of cardiovascular outcomes, murine models may exacerbate adaptive responses to hypoxia (Campen et al., 1985; Naghshin et al., 1985). It is possible that the differences in our findings from those of other studies are due to differences in the IH protocol, including the oxygen concentrations used and the pattern and frequency of hypoxia (Dale et al., 2014; Navarrete‐Opazo & Mitchell, 2014). Finally, several studies have demonstrated important acute effects of IH that could impair some biomarkers at the time of euthanasia (Bernaudin et al., 2002; Crosson et al., 2009; Hassan et al., 2018; Manella et al., 2020). Models of acute exposure and recovery from to IH are needed to elucidate early changes in *HIF*‐*1* and *VEGF* expressions.

In the present study, the Matrigel plug was surrounded by inflammatory cells. VEGF and interleukin 8 may be produced by monocytes/macrophages, consequently inducing angiogenesis in the heart (McSweeney et al., 2010). However, even this possible angiogenic response to inflammation was reduced in the IH model relative to normoxic animals. We are aware that angiogenesis is a multifactorial and complex event involving a great number of genes and controls. We have not addressed many other factors, such as PDGF, PlGF, and angiopoietins (Gogiraju et al., 2019), that could be altered in this model. Also, little is known about the mechanism underlying the lack of correlation between *ACE* and *CPM* mRNA levels and corresponding protein activities during IH exposure. Mice express 33 different non‐coding *CPM* RNAs in a tissue‐specific manner (Guimaraes et al., 2009), indicating a critical influence of post‐transcriptional gene regulation that may affect protein translation and activity (Zhang et al., 2019). Finally, it is essential to consider that *B2R* is constitutively expressed, whereas *B1R* is unexpressed in normal conditions and is upregulated following exposure to lipopolysaccharides, tissue injury, myocardial infarction, or anoxia (Kuhr et al., 2010). Considering that B1R is upregulated by B2R activation, we should expect an increase in B1R expression during IH. The discrepancy of our results with this expectation might be explained by the transience of the increase in CPM activity, that enzyme being necessary to convert B2R agonist to B1R agonist. Furthermore, reduction of B1R downregulates CPM expression and activity in endothelial cells (Guimaraes et al., 2019). We were able to quantify *B1R* expression using RT‐PCR; however, we could not document the aforementioned B1R downregulation through pharmacological approaches, which would be relevant and should be considered in future studies.

## 5 CONCLUSIONS

The present study reports that under IH, myocardium angiogenesis and capillary density are reduced while cardiac hypertrophy, the number of EPCs, B2R expression, and angiogenic factors are increased. Taken together, the delayed increase of VEGF_120_, VEGF_164_, and its receptor; the transient increase in CPM activity; and the reduction of *B1R* expression indicate impaired angiogenesis. Our results indicate a potential role of reduced capillary density and impaired angiogenesis in the hypertrophic heart, with potential implications in heart malfunction. These observations could be essential to understanding the development of myocardial hypertrophy and progression of cardiovascular complications in patients with sleep apnea. With regard to prior reports on adaptive cardioprotective responses in hypoxia, we cannot exclude the possibility that out observation of impaired angiogenesis might be due to differences in the severity, duration, and pattern of IH. Nonetheless, we can expect that the classic interaction of the VEGF system with the KKS, an interaction essential for angiogenesis under hypoxic conditions, is deeply disturbed in IH, which potentially explains the observed impairment of myocardial angiogenesis. These results suggest that in IH, unlike in other models of hypoxia, the classic VEGF–KKS interaction is deficient in stimulating the formation of new vessels.

## DISCLOSURE STATEMENTS

No authors declare conflict of interests.

## AUTHOR CONTRIBUTIONS

BV and JCR designed, conducted, and performed all the experiments, data analysis, and wrote the manuscript. JCP designed the experimental protocol and data analysis. ANP and EJPG performed the flow cytometry. GGO performed the immunohistochemistry. JCP, EJPG, GGO, ST, and JCR provided critical revisions of the study and manuscript. All authors reviewed the manuscript.

## Supporting information



Supplementary Material
