# Supplementary material for: Intermittent hypoxia changes the interaction of the kinin–VEGF system and impairs myocardial angiogenesis in the hypertrophic heart
Source: Physiol Rep. 2021 May 15;9(9):e14863. doi: 10.14814/phy2.14863 (PMC8123545; doi:10.14814/phy2.14863)
Supplement: Supplementary file 1 — Supplementary Material [file PHY2-9-e14863-s001.docx]

**Supplementary Information**

**Intermittent hypoxia changes the interaction of the kinin-VEGF system and impairs myocardial angiogenesis in the hypertrophic heart**

Bruna Visniauskas^1^, Juliana C. Perry^1^, Guiomar N. Gomes^2^, Amanda Nogueira-Pedro^3^, Edgar J. Paredes-Gamero^3^, Sergio Tufik^1^, Jair R. Chagas^1,3^**^*^**

^1^Departamento de Psicobiologia, Universidade Federal de São Paulo, São Paulo, Brazil

^2^Departmento de Fisiologia, Universidade Federal de São Paulo, São Paulo, Brazil

^3^Departamento de Biofísica, Universidade Federal de São Paulo, São Paulo, Brazil.

**Address for Correspondence:**

Jair Ribeiro Chagas

Department of Biophysics

Universidade Federal de São Paulo

Rua Botucatu, 862 - 7o andar – CEP: 04023-062

São Paulo, SP/ Brasil.

Tel.: +55 (11) 5576-4848 Ext. 2342/2343

Fax: +55 (11) 5571-5780

Email: jchagas1@gmail.com


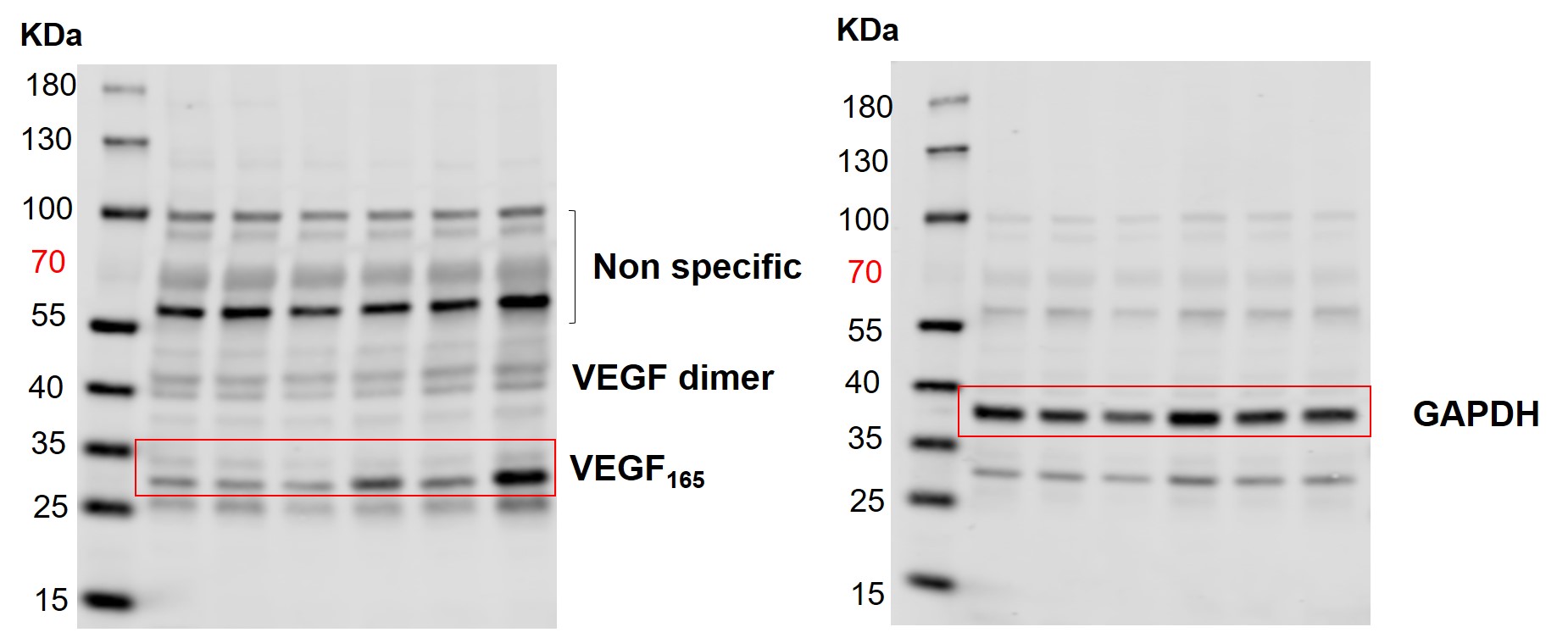

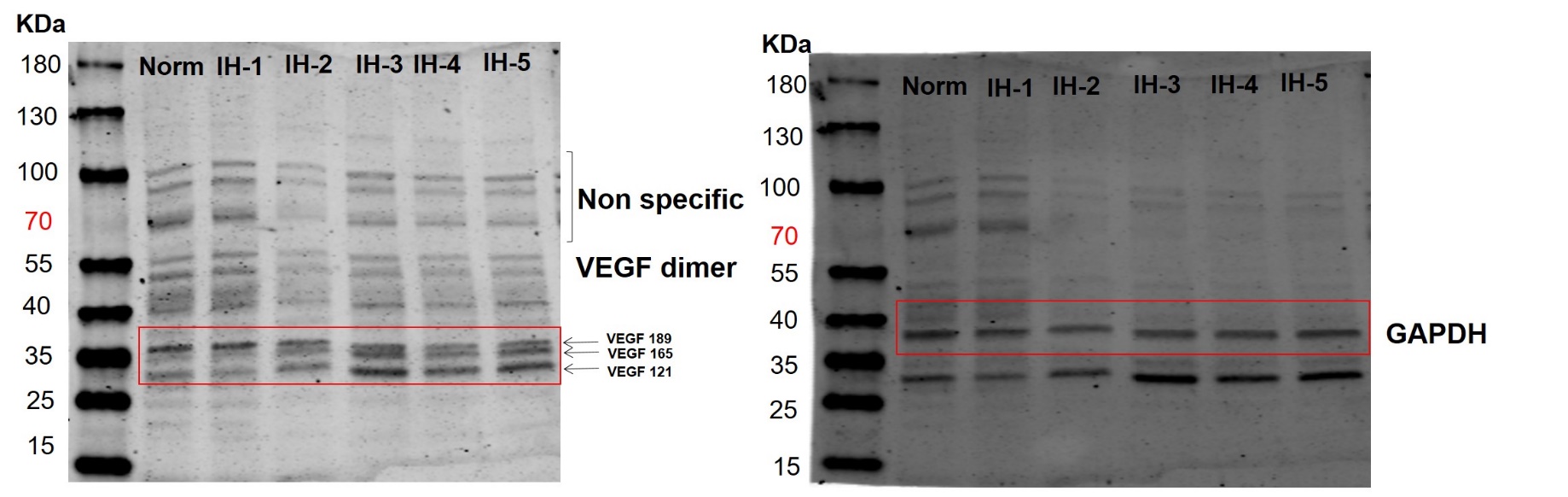
**Full length Western Blots used in Figure 6.**

According to the company the predicted VEGF molecular weight is 27 kDa. The reference band of 70 kDa stained in orange reference was not apparated using Alexa-Fluor 680 (red filter) in Odyssey Licor System. After anti-VEGF incubation, the membranes were then stripped and re-probed with Tris-HCl 68.5 mM, pH 6.8 with SDS 2%, β-mercaptoethanol 100 mM buffer for 30 minutes at 58 °C

**Full length Western Blots used in Figure 7 and anti-B2R KO validation.**


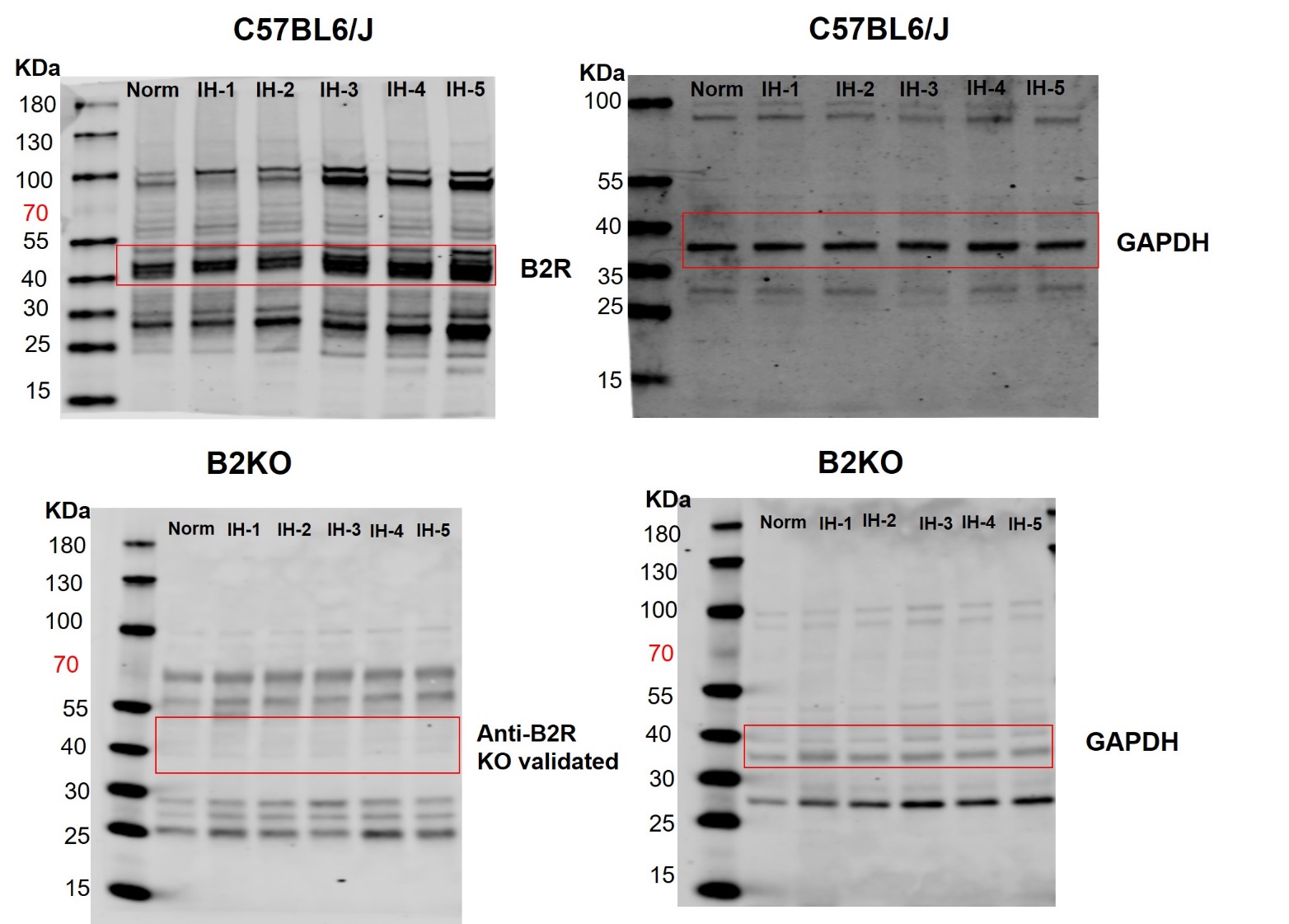


The anti-B2R (Proteimax Co, Brazil) was validated in heart tissue of global knockout mice. According to the Uniprot database the predicted B2R molecular weight is 44 kDa. In prestained protein molecular weight marker, the reference band of 70 kDa stained in orange reference was not apparated using Alexa-Fluor 680 (red filter) in Odyssey Licor System.
